# Dosimetric analysis of radiation-induced brainstem necrosis for nasopharyngeal carcinoma treated with IMRT

**DOI:** 10.1186/s12885-022-09213-z

**Published:** 2022-02-17

**Authors:** Xigang Fan, Yecai Huang, Peng Xu, Yanmei Min, Jie Li, Mei Feng, Guohui Xu, Jinyi Lang

**Affiliations:** 1 Department of Oncology, People’s Hospital of Deyang City, Deyang, Sichuan China; 2 Department of Radiation Oncology, Radiation Oncology Key Laboratory of Sichuan Province, Sichuan Cancer Hospital & Institute, Sichuan Cancer Center, Chengdu, Sichuan China; 3 School of Medicine, University of Electronic Science and Technology of China, Chengdu, Sichuan China; 4 Department of Oncology, The Third Hospital of Mianyang, Mianyang, Sichuan China; 5 Department of Interventional Radiology, Sichuan Cancer Hospital and Institute, Sichuan Cancer Center, Chengdu, Sichuan China

**Keywords:** Brainstem necrosis, Tolerance dose, Nasopharyngeal carcinoma, Intensity-modulated radiotherapy

## Abstract

**Background:**

Radiation-induced brainstem necrosis (RIBN) is a late life-threatening complication that can appear after treatment in patients with nasopharyngeal carcinoma (NPC). However, the relationship between RIBN and radiation dose is not still well-defined.

**Methods:**

During January 2013 and December 2017, a total of 1063 patients with NPC were treated at Sichuan cancer hospital with IMRT. A total of 479 patients were eligible for dosimetric analysis. Dosimetric parameters of the RIBN, D_max_(the maximum dose), D_0.1c_ (maximum average dose delivered to a 0.1-cc volume), D_1cc_, D_2cc_, D_3cc_, D_5cc_, D_10cc_ and D_mean_ (mean does) were evaluated and recorded. ROC curve was used to analyze the area under curve (AUC) and cutoff points. Logistic regression for screening dose-volume parameter and logistic dose response model were used to predict the incidence of brainstem necrosis.

**Results:**

Among the 479 patients with NPC, 6 patients were diagnosed with RIBN, the incidence of RIBN was 1.25% (6/479), and the median time to RIBN after treatment was 28.5 months (range 18–48 months). The dose of the brainstem in patients with RIBN were higher than that in patients without necrosis. ROC curve showed that the area under the curve (AUC) of D_max_ was the largest (0.987). Moreover, logistic stepwise regression indicated that D_max_ was the most important dose factor. The RIBN incidence at 5% over 5 years (TD_5/5_) and 50% incidence over 5 years (TD_50/5_) was 69.59 Gy and76.45 Gy, respectively.

**Conclusions:**

Brainstem necrosis is associated with high dose irritation. D_max_ is the most significant predictive dosimetric factor for RIBN. *D*_max_ of brainstem should be considered as the dose limitation parameter. We suggest that the limitation dose for brainstem was D_max_ < 69.59 Gy.

## Introduction

Nasopharyngeal carcinoma (NPC) is a common malignancy in southern China, with an incidence of 5.38–11.16 per 100,000 people [1, 2]. Radiotherapy or chemoradiotherapy is currently considered as the basic treatment for NPC [2], and the tumor local-control is positively associated with the total dose of irradiation [3, 4]. Intensity-modulated radiotherapy (IMRT) can lead to acceptable disease control and fewer complications. The current 5-year and 10-year overall survival rate of NPC treated with IMRT is 77.1–82.6% [5–7] and 49.5% [3], respectively.

However, with the prolonged survival time after radiotherapy, more and more late complications are worthy of attention. For example, in patients with long-term survival, radiation-induced nerve injury could directly influence the quality of life [8]. In addition, radiation-induced brain necrosis(RIBN) is a late life-threatening complication, which can appear after treatment. When using 2-dimensional (2D) conformal radiotherapy, RIBN occurs in 5.5% of patients, with a mortality rate of 0.9% [9]. Yet, several studies have reported that the incidenceof RIBN can be significantly reduced when using IMRT. For example, a retrospective study showed that only 0.13–2.8% [10–12] of patients with NPC developed brainstem necrosis after IMRT. RIBN is a late complication with low incidence but still worth of attention.

In the IMRT era, radiotherapist are usually confused by the tolerance dose of the brainstem, while the dose limit of conformal radiotherapy for the brainstem has been well-established [13]. Huang et.al reported a total of 24 BSI in 6288 patients who underwent IMRT and suggested a Dmax of 67.4 Gy (D2) as the dose constraint for brain stem [12]. However, the author also admitted that this was not ideal because it had relatively poor positive predictive value [12]. Thus, we obtained the dose-volume data for organ at risk(OAR) from the treatment planning system and re-evaluated the tolerance dose of brainstem in IMRT era based on data in our institution.

## Materials and Methods

### Patients

A total of 1063 NPC patients who received IMRT at our hospital between January 2013 and December 2017 were considered for this study. The inclusion criteria for dosimetric analysis were: (1) pathologically confirmed NPC; (2) no distant metastasis; (3) patients undergoing radical whole-course IMRT; (4) without other head and neck malignancies. The exclusion criteria were: (1) received re-irradiation for local recurrence; (2) follow-up time < 5 years; (3) without regular follow-up after MRI; (4) among the 1063 NPC patients, 533 patients with < 60 months follow-up and 52 relapsed patients who received IMRT re-irradiation were excluded from the dosimetric analysis. Finally, 479 patients were eligible for the final dosimetric analysis (Table 1). This study was approved by the Ethics Committee of Sichuan Cancer Hospital. Written informed consent was obtained from all patients. Patients were treated in accordance with the Radiation Therapy Oncology Group protocol 0225 (RTOG0225)and our institutional guidelines.

**Table 1:** Characteristics of 479 patients, n (%)

		Normal(*N* = 473)	Necrosis(*n* = 6)	Total(479)
Age		45.78 ± 11.39	43.83 ± 12.19	45.73 ± 11.39
Gander	Femal	116(24.5)	2(33.3)	118(24.6)
	Male	357(75.5)	4(66.7)	361(75.4)
T Classification	Stage T1	44(9.3)	0 (0.00)	44 (9.2)
	Stage T2	107 (22.6)	0 (0.00)	107(22.3)
	Stage T3	145 (30.9)	1 (16.7)	146 (30.5)
	Stage T4	177(37.4)	5 (83.3)	182 (38.0)
Stage	I	3 (0.6)	0 (0.00)	3 (0.6)
	II	54(11.4)	0 (0.00)	54(11.3)
	III	226(47.8)	1 (16.7)	227(47.4)
	IV	190(40.2)	5 (83.3)	195(40.7)
D01	≤ 60 Gy	441(93.2)	0(0.0)	441(92.1)
	> 60 Gy	32(6.8)	6(100.0)	38(7.9)

### Clinical staging

All patients underwent physical examination, endoscopy, head and neck MRI, chest radiography, and dental assessment, and were restaged according to the eighth American Joint Committee on Cancer staging system (AJCC, 2017).

### Treatment

#### Radiotherapy

The patient was placed in a supine position. A thermoplastic mask was applied, and a 2.0 cm cork was affixed to the mouth. Computed tomography (CT) with contrast was applied for the treatment planning. The scanning slice thickness was 3.0 mm, with a 2.5-mm slice gap. The CT slices ranged from the top of the head to the level of the sternoclavicular joint. T1-weighted and T2-weighted fast spin-echo images, and T1-weighted imaging with intravenous administration of gadopentetate dimeglumine were acquired for image fusion and contouring.

IMRT treatment strategy was optimized with an inverse planning system (Peacock, Nomos, Deer Park, IL, USA, version 3.4–4.2), and MiMi multileaf collimator (Nomos, Sewickly, PA, USA). The gross tumor volumes (GTVs) were determined according to the guidelines of the International Commission on Radiation Units and Measurements, report 50 [14] and 62 [15]. As determined by clinical evaluation, endoscopy, CT and MRI, the borders of the primary nasopharyngeal tumor and the involvement of lymph nodes were determined, and the GTVs of the nasopharynx and bilateral positive neck lymph nodes were contoured.

Clinical target volume (CTV) 1 was defined as an isotropic expansion of the GTV of the nasopharynx, with a 5–10 mm margin. CTV2 included CTV1 and the following high-risk local structures: parapharyngeal space, posterior third of nasal cavity and maxillary sinus, pterygoid process, skull base, lower half of the sphenoid sinus, anterior half of the clivus,and the petrous tip. The CTV of the lymph nodes was defined as the lymphatic drainage regions, where the margins were broadened by 3 mm due to uncertainty. The prescribed doses for each target were as follows: GTV of the nasopharynx, 66–76 Gy; GTV of the lymph nodes, 60–70 Gy; CTV1, 60–66 Gy; CTV2, 55–60 Gy; and CTV of the lymph nodes, 50–55 Gy in 30-36fractions. Cobalt-60 split-field technique or 6-MV X-ray split-beam technique were applied to lymph node drainage areas in the lower neck at 50 Gy for 25 fractions. The dose limits for organs at risk in the first course were in accordance with the Radiation Therapy Oncology Group 0225 (RTOG 0225). In particular, the dose limit for the brainstem was D_01_ (dose to 1% of brainstem volume) ≤ 60 Gy [16]. When prescribing a radical dose to GTV, the informed consent form was re-written for patients whose (OAR) might receive higher dose than the RTOG 0225.

#### Chemotherapy

Among the 479 patients, 442 received cisplatin-based chemotherapy with docetaxel (75 mg/m^2^) + cisplatin (75–80 mg/m^2^)(TP) or cisplatin (80–100 mg/m^2^). Meanwhile, 123 patients received induction chemotherapy with docetaxel (60 mg/m^2^) + cisplatin (60 mg/m^2^) + fluorouracil (600 mg/m^2^)(TPF) on days 1–5, TP or gemcitabine (1.0 g/m^2^) on days 1 and 8 + cisplatin (75–80 mg/m^2^) on days 1, for 2–3 cycles before concurrent chemotherapy. Additionally, 43 patients received cetuximab and 40 received nimotuzumab therapy.

### Brainstem delineation and dose analysis

The brainstem was delineated based on the MRI and CT fusion images. The superior boundary was the mammillary body and posterior commissure; the inferior boundary was the posterior rim of the foramen magnum [17]. To determine the dose that the brainstem received, the original plan and CT/MRI images were obtained, and the brainstem was delineated according to the original outline of the copied plan. The dose used for statics was calculated on “pv-brainstem” which was based on the volume with 2 mm PRV expansion from the original outline of brainstem. A validation plan with the re-contoured brainstem was finalized, from which Dmax, D_0.1 cc_, D_1cc_, D_2cc_, D_3cc_, D_5cc_, D_10cc_ and D_mean_ of the brainstem were calculated. The equivalent dose is the 2 Gy fractions of the brainstem, calculated by the following equation:

$$D2=Dx\times (\alpha /\beta +dx)/(\alpha /\beta +2)$$

(Dx = total physical dose, dx = fraction dose;α/βvalue of brainstem was 2.1 [18])

### Follow-up

Follow-ups were conducted every three months for the first two years, then every six months for three years, and annually after the fifth year. Disease status and treatment toxicities were assessed using head and neck MRI, chest radiography, abdominal ultrasound, physical examination (each time), and whole-body bone scanning(annually) by physicians. The duration of the follow-up was considered as the time after IMRT to either brainstem necrosis occurrence or the last follow-up. The follow-up deadline was December 2019. Radiation-induced toxicities and late toxic reactions were graded in accordance with the Common Terminology Criteria for Adverse Events version 4.03 [19].

### Toxicity diagnostic criteria

**Fig. 1:**
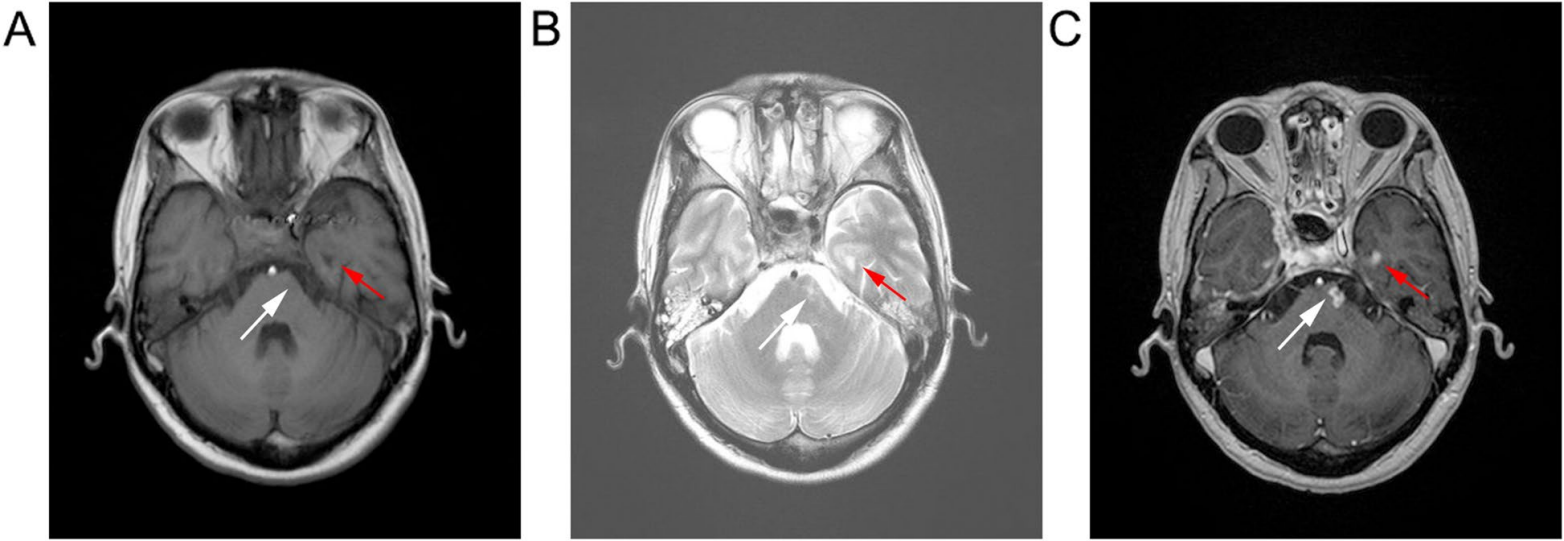
Typical presentation of brainstem necrosis on MRI of a 43-year-old man, treated with IMRT. Axial images showing marginal enhancement and surrounding edema in the pons (white arrows) and left temporal lobe (red arrows). **a** T1-weighted image; **(b)** T2-weighted image; and **(c)** contrast-enhanced T1-weighted image. The patient had a mild memory loss and swallowing dysfunction at 42 months of follow up

The diagnostic criteria for brainstem necrosis were based on contrast enhancement on T1-weighted images and heterogeneous hyper intensity on T2-weighted images. The region of necrosis could be surrounded by an area of edema, indicated by homogeneous hyper intensity on T2-weighted MR images (Fig. 1) [20, 21]. All images were independently reviewed by two senior radiologists with more than 10 years experience. A diagnosis of brainstem necrosis was decided by consensus if there were different diagnostic opinions.

Brainstem injury was graded based on symptoms in accordance with the recommendations of Cancer Therapy Evaluation Program [19]: Grade 1, mild or asymptomatic; Grade 2, moderate without interfering with the daily life activities; Grade 3, severe effect on the daily life activities, intervention may be required; Grade 4, life-threatening or disabling, intervention required; and Grade 5, death.

### Statistical analysis

All statistical analyses were performed using SPSS version 20.0 software (IBM, Armonk, NY, USA). If continuous variables conformed to normal distribution, they were expressed as mean ± standard deviation (SD); the comparison between the two groups was analyzed using independent t-test. If they did not conform to normal distribution, they were expressed as median (range); and the comparison between the two groups was analyzed by non-parametric test, Chi-square test. Significant dosimetric parameters were detected by receiver operating characteristic (ROC) curve and stepwise logistic regression. Prediction model for significant dosimetric factors was established using logistic dose response model. A *p-*value < 0.05 was considered to be statistically significant.

## Results

The median age of the 479 patients was 45.73 ± 11.39 years; the ratio of male and female was 2.99:1; and the median follow-up duration was 61 months (range 18–84 months). Patients in stages T3 and T4 accounted for 68.48% (*n* = 328) of all patients included in the dosimetric analysis.

Among 38 patients who were at risk of having brainstem necrosis (as brainstem D01 > 60 Gy) (Table 1), Six patients developed brainstem necrosis (Table 2, Fig. 1),giving a crude incidence of 15.7% for those patients with brainstem dose constraint exceeding the RTOG recommendation. The median time to necrosis was 28.5 months (range 18–48 months). The brainstem necrosis occurred only in patients with locally advanced T3 (1) and T4 (5) stages. Three out of six patients experienced medulla oblongata necrosis, while necrosis occurred in the pons or pons plus medulla oblongata in five cases. In addition, among the six patients with brainstem necrosis, one, two, and one patients experienced grade 2, 3, and 4 toxicity, respectively. Two patients experienced grade five toxicity. Five patients experienced impaired motor function, three experienced fatigue, three experienced paresthesia; two patients suffered paralysis, and one patient experienced swallowing dysfunction. Consequently, all six patients with necrosis died (three due to distant metastasis, two due to complications (pneumonia), and one due to nasopharyngeal hemorrhage).

**Table 2:** Characteristics of the 6 patients with brainstem necrosis after IMRT

	Gender	Age, y	Stage	Necrosis time, mo	Necrosis location	D_max_, Gy	Grade
1	Female	56	T4N2M0	20	Pons + medulla	80.66	5
2	Male	43	T4N2M0	42	Pons	77.38	5
3	Male	30	T4N1M0	48	Pons	75.32	4
4	Male	61	T3N2M0	24	Medulla	71.08	3
5	Male	36	T4N2M0	33	Pons + medulla	71.00	3
6	Female	37	T4N1M0	18	Pons	71.58	2

### Dosimetric analysis

**Fig. 2:**
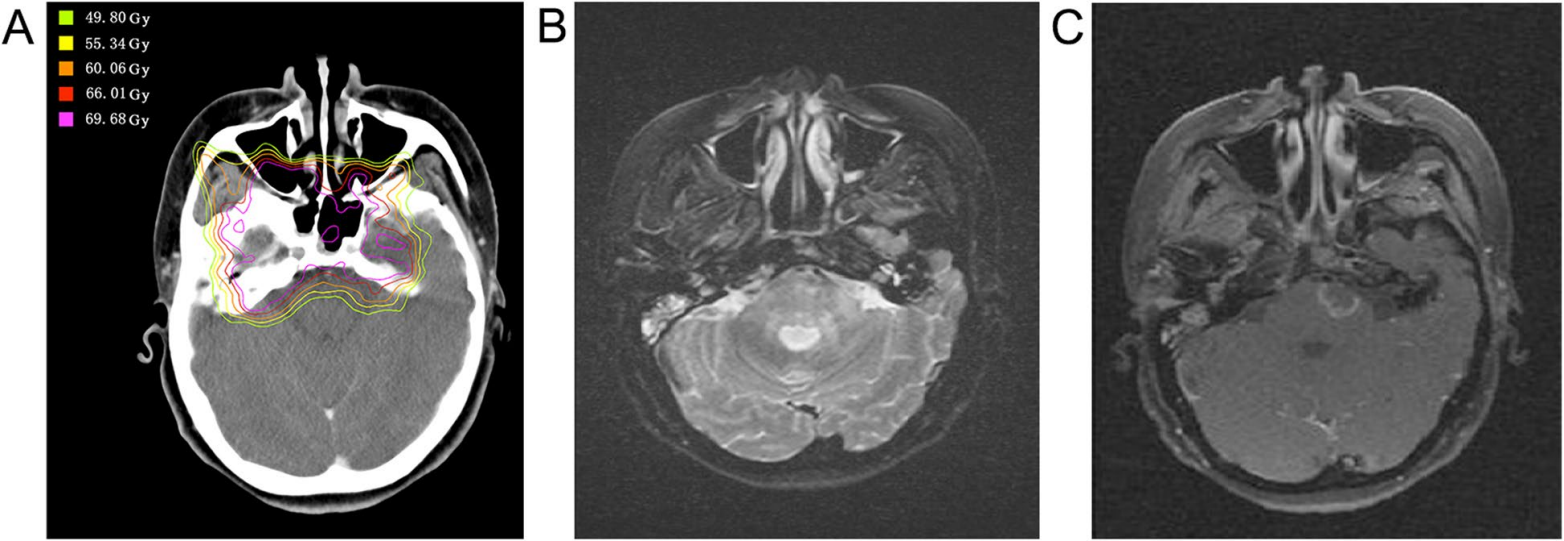
Typical presentation of brainstem necrosis on MRI of a 37-year-old woman, 18 months after IMRT. Significant edema and irregular enhancement in the high-dose area of the left pons was observed. **a** IMRT dose distribution around the brainstem, in which the max dose was located in the left front of pons; **(b)** T2-weighted image; and **(c)** contrast-enhanced T1-weighted image

The six cases with brainstem necrosis received high dose brainstem exposure. The *D*_max_ and high dose regions were located on the anterior and lateral surfaces of the brainstem. Necrotic lesions were located in the high dose areas. In addition, no hotspot was found at the center of the brainstem (Fig. 2). Moreover, necrotic brainstem received higher X-ray irradiation. D_max_, D_0.1 cc_, D_1cc_, D_2cc_, D_3cc_, D_5cc_, D_10cc_ and D_mean_ of the brainstem in patients with brainstem necrosis were higher than those of patients without necrosis (Table 3).

**Table 3:** The dose of necrosis and non-necrosis brain stem

Dosimetric factor	Non-brainstem necrosis		Brainstem necrosis		*p*-value
	Mean	SE	Mean	SE	
D_max_	48.77	10.71	74.50	10.71	0.00
D_0.1 cc_	43.44	10.62	68.28	6.05	0.00
D_1cc_	35.87	9.60	56.62	7.25	0.00
D_2cc_	31.86	8.97	50.94	9.10	0.00
D_3cc_	29.54	8.41	47.23	10.39	0.00
D_5cc_	25.94	7.58	41.56	11.33	0.00
D_10cc_	20.16	6.29	32.48	11.39	0.00
D_mean_	14.35	5.02	23.28	8.75	0.00

### Significant dosimetric factors

The ROC curve analysis indicated that eight dosimetric factors were significantly associated with brainstem necrosis (Table 4). Dmax showed a maximum area under curve (AUC:0.987, *p* = 0.000).

**Table 4:** ROC curve analysis for dosimetric factors

Dosimetric factor	Area under curve	SE	*p*-value
_max_	0.987	0.006	0.000
D_0.1 cc_	0.966	0.017	0.000
D_1cc_	0.951	0.019	0.000
D_2cc_	0.934	0.026	0.000
D_3cc_	0.918	0.034	0.000
D_5cc_	0.900	0.045	0.001
D_10cc_	0.839	0.089	0.004
D_mean_	0.813	0.111	0.008

In the univariate analysis, all eight dosimetric factors were significantly associated with brainstem necrosis (Table 5). Multivariate logistic regression analysis to predict brainstem necrosis showed that *D*_max_ was the only significantly dosimetric factor associated with RIBN. The odds ratio(OR) of *D*_max_ was 1.91 (95% confidence interval(CI): 1.09,3.32, *p* = 0.02).

**Table 5:** Univariate and multivariate analysis for dosimetric factors

Dosimetric factors	Univariate analysis			Multivariate analysis		
	Odds ratio	95%CI for HR	p-valve	Odds ratio	95%CI for HR	*p*-valve
D_max_	1.38	1.15,1.67	0.00	1.91	1.09,3.32	0.02
D_0.1 cc_	1.28	1.11,1.47	0.00	0.88	0.55,1.43	0.61
D_1cc_	1.20	1.09,1.32	0.00	0.31	0.29,1.48	0.31
D_2cc_	1.18	1.09,1.29	0.00	1.05	0.64,1.73	0.85
D_3cc_	1.17	1.08,1.28	0.00	1.27	0.42,3.37	0.18
D_5cc_	1.17	1.08,1.266	0.00	0.90	0.01,2.18	0.14
D_10cc_	1.19	1.09,1.29	0.00	2.39	0.76,7.49	0.14
D_mean_	1.26	1.11,1.42	0.0	1.19	0.67,2.13	0.55

**Fig. 3:**
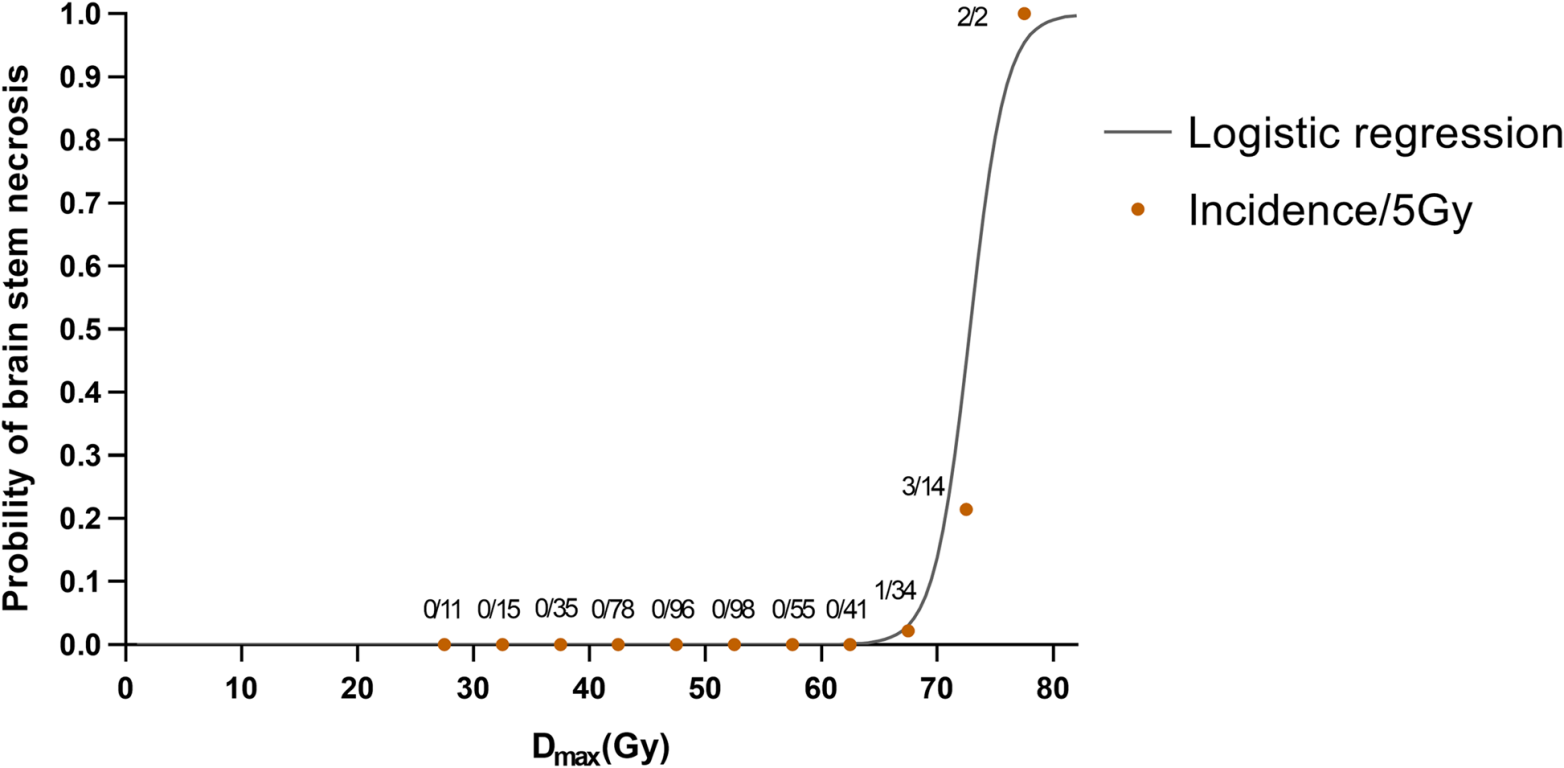
Dose response curves of probability of brainstem necrosis. The longitudinal axis is the predictive incidence of brainstem necrosis. The incidence of brain necrosis in the 5-Gy interval is expressed as the number of cases per total number of cases in the 5-Gy dose range. The dots in the graph represent the average of the dose range

Dmax was included into the logistic regression model for brainstem necrosis dosimetric analysis. Briefly, the tolerance dose (TD) that would result in1% (TD_1/5_), 5% (TD_5/5_), 10%, 30%, and 50% risk of brainstem necrosis within five years after IMRT was 65.8 Gy, 69.59 Gy, 71.50 Gy, 74.48 Gy, and 76.45 Gy, respectively (Fig. 3).

## Discussion

RIBN is a life-threatening complication. In patients with locally advanced NPC, the irradiation dose to tumor is closely associated with the tolerance dose of normal tissue. The dose determination depends on the tradeoff between tumor coverage and the risk of brainstem complications [12, 22].The median time to injury was 28.5 months (range 18–48 months) in this study, which was a little longer that 21 months reported in other studies [11].The latency of RIBN in the modern RT era is slightly longer than that in the conventional RT era, ranging from 6 months to 2 years [23].Clinical factors such as host-related (ie, age, smoking, drinking, hypertension, diabetes), tumor-related (ie,tumor stage), and treatment-related factors (ie, chemo-therapy, radiation technology) were associated with RIBN [24]. Locally advanced NPC (T3 or T4) accounted for 68.48% of all newly detected cases. All patients who experienced RIBN were in T3(*n* = 1)and T4(*n* = 5)stages, which was consistent with the results reported by Yao et al. and Huang et al. [11]. With the emergence of IMRT and more precision radiotherapy equipment in the future, the occurrence of RIBN will be reduced significantly. The brainstem injury rate in this study was lower than the rate (3.8–5.5%) associated with 2D-conformal radiotherapy [9, 25].

According to RTOG 0225,0615 and QUANTEC, a maximum dose of 54 Gy to the brainstem was ideally recommended. What’s more, an acceptable alternate was to allow < 60 Gy to 1% of the brainstem volume [16, 26]; In conventional conformal radiotherapy, Emami et al*.* [27] showed that with a 5% brainstem necrosis risk, the tolerance dose for one-third, two-third, and the entire brainstem, was 60, 53, and 50 Gy,respectively. According to the Lyman-Kutcher-Berman calculation, there is a 50% probability of complications within five years for entire brainstem when the radiation dose is 65 Gy. Mayo et al*.* [13] found that the maximum tolerance irradiation dose for the total brainstem was 54 Gy when using 3-dimensional conformal radiation therapy, and the tolerance dose of a small volume of the brainstem (≤ 10 cc) was 59 Gy. There was a significant increase in brainstem injury at doses > 64 Gy.

Few studies focus on the tolerance dose of brainstem injury in the IMRT era. Li et al*.* [10] reported that D_max_, D_1cc_, D_2cc_, aV_50_, aV_55_ and aV_60_ were the limited dosimetric factors, while Yao and his team [11] reported that D_max_, D_1%_, D_0.1 cc_ and D_1cc_ were the limited dosimetric factors. The largest sample study till now was published in 2019, which showed that the tolerance dose for brainstem should be constrained to Dmax < 67.2 Gy(D2) [12]**.** Our study analyzed the dose characteristics, dosimetric predictive factors and dose–response relationship of brainstem necrosis in patients with NPC. Our findings suggested that the TD_5/5_of brainstem should be Dmax = 69.59 Gy (D2) based on the NTCP model we used. Paticipants with either edematous lesions or contrast-enhanced lesions in the paper published by Huang et.al were included as “brainstem injury” patients. In our study, only those with contrast-enhanced lesions were included and defined as “brainstem necrosis” patients. We knew that necrosis was more serious than edema and was usually induced by higher radiation dose. Study the difference in inclusion criteria and study purposes might explain the reason why the tolerance dose of “brainstem necrosis” we suggested was higher than tthat of “brain stem injury” reported by Huang et.al [12].

*D*_max_ is a common dosimetric parameter that is widely used in clinical practice to evaluate RIBN [28–30]. Like other serial element models, brainstem injury depends on functional subunits [31, 32]. A small volume injury can cause serious complications. Moreover serial organ damage is associated with high doses in small volumes, Dmax could be used as a predictive dosimetric factor,which was consistent with their conclusions [24].

This study found that the tolerance dose with a 5% risk of brainstem necrosis delivered by IMRT within five years (TD_5/5_) was *D*_max_69.59 Gy, which was higher than that reported by another study [13]. Debus et al*.* [9] found that the brainstem toxicity-free survival at 10-year was 96% in patients who received a brainstem tolerance dose of V60 < 0.9 cc. Moreover, Li et al. reported that 24.9% (1544) of patients had a *D*_max_ > 64 Gy, of which only two patients experienced RIBN [10]. Furthermore, Yao et al. reported a D_max_ of 67.85 Gy [11], which was consistent with our conclusion. These results may be ascribed to the advantages of IMRT, including the dose gradient alteration and hotspot of the brainstem. The target area in a NPC treatment plan is close to many vital organs. IMRT provides better dose accuracy compared with conformal radiotherapy, and the hotspots are limited to the brainstem surface. In this study, the hotspot was on the surface of the brainstem and the dose distribution conformed to the anatomical contour. Combined with MRI fusion, which could facilitate correct contouring of the brainstem, the incidence of RIBN was low. Given the large number of NPC patients who are treated with IMRT every year, the incidence rate of brainstem necrosis is relatively low. Among patients with cranial invasion, the tolerance dose to the brainstem of NPC patients may vary, probably because the high dose region is close to the surface of the brainstem (mainly anterior and lateral), rather than at the center of the brainstem.

This study had a few limitations. First, the included studies were characterized with small sample sizes (limited number of cases of RIBN). Second,few dose-volume parameters and other factors such as (diabetes, and hypertension) were considered. Thus, more clinical trials should be performed to further examine the brainstem tolerance doses in patients with NPC.

This retrospective analysis showed that in patients with NPC, brainstem necrosis was significantly associated with the IMRT radiation dose. Dmax was the most important predictive dosimetric factor. In the a conventional fractionation scheme, the tolerable dose of the brainstem was Dmax, and its value should not exceed 69.59 Gy(D2).

## Data Availability

The datasets used and analysed during the current study available from the corresponding author on reasonable request.
